# PaCO_2 _and alveolar dead space are more relevant than PaO_2_/FiO_2 _ratio in monitoring the respiratory response to prone position in ARDS patients: a physiological study

**DOI:** 10.1186/cc10324

**Published:** 2011-07-25

**Authors:** Cyril Charron, Xavier Repesse, Koceïla Bouferrache, Laurent Bodson, Samuel Castro, Bernard Page, François Jardin, Antoine Vieillard-Baron

**Affiliations:** 1 Intensive Care Unit, Section Thorax-Vascular Disease-Abdomen-Metabolism, Ambroise Paré University Hospital, AP-HP, 9 Av Charles de Gaulle, F-92104 Boulogne-Billancourt Cedex, France; 2 Faculté de Paris Ile-de-France Ouest, Université de Versailles Saint Quentin en Yvelines, 9 boulevard d'Alembert, F-78280 Guyancourt, France

## Abstract

**Introduction:**

Our aims in this study were to report changes in the ratio of alveolar dead space to tidal volume (VD_alv_/V_T_) in the prone position (PP) and to test whether changes in partial pressure of arterial CO_2 _(PaCO_2_) may be more relevant than changes in the ratio of partial pressure of arterial O_2 _to fraction of inspired O_2 _(PaO_2_/FiO_2_) in defining the respiratory response to PP. We also aimed to validate a recently proposed method of estimation of the physiological dead space (VD_physiol_/V_T_) without measurement of expired CO_2_.

**Methods:**

Thirteen patients with a PaO_2_/FiO_2 _ratio < 100 mmHg were included in the study. Plateau pressure (Pplat), positive end-expiratory pressure (PEEP), blood gas analysis and expiratory CO_2 _were recorded with patients in the supine position and after 3, 6, 9, 12 and 15 hours in the PP. Responders to PP were defined after 15 hours of PP either by an increase in PaO_2_/FiO_2 _ratio > 20 mmHg or by a decrease in PaCO_2 _> 2 mmHg. Estimated and measured VD_physiol_/V_T _ratios were compared.

**Results:**

PP induced a decrease in Pplat, PaCO_2 _and VD_alv_/V_T _ratio and increases in PaO_2_/FiO_2 _ratios and compliance of the respiratory system (Crs). Maximal changes were observed after six to nine hours. Changes in VD_alv_/V_T _were correlated with changes in Crs, but not with changes in PaO_2_/FiO_2 _ratios. When the response was defined by PaO_2_/FiO_2 _ratio, no significant differences in Pplat, PaCO_2 _or VD_alv_/V_T _alterations between responders (*n *= 7) and nonresponders (*n *= 6) were observed. When the response was defined by PaCO_2_, four patients were differently classified, and responders (*n *= 7) had a greater decrease in VD_alv_/V_T _ratio and in Pplat and a greater increase in PaO_2_/FiO_2 _ratio and in Crs than nonresponders (*n *= 6). Estimated VD_physiol_/V_T _ratios significantly underestimated measured VD_physiol_/V_T _ratios (concordance correlation coefficient 0.19 (interquartile ranges 0.091 to 0.28)), whereas changes during PP were more reliable (concordance correlation coefficient 0.51 (0.32 to 0.66)).

**Conclusions:**

PP induced a decrease in VD_alv_/V_T _ratio and an improvement in respiratory mechanics. The respiratory response to PP appeared more relevant when PaCO_2 _rather than the PaO_2_/FiO_2 _ratio was used. Estimated VD_physiol_/V_T _ratios systematically underestimated measured VD_physiol_/V_T _ratios.

## Introduction

Since its first description in 1967 [1], it has been accepted that acute respiratory distress syndrome (ARDS) includes a number of lung injuries of various origins whose consequences are decreased lung capacity available for ventilation, leading to the concept of "baby lung" [2]. Considerable progress has been made over the past decade in the ventilatory management of patients with ARDS. In particular, a strict limitation of tidal volume (V_T_) and plateau pressure (Pplat) below 30 cmH_2_O reduces mortality [3]. The application of positive end-expiratory pressure (PEEP) is recognized to recruit the lung and to restore functional residual capacity [4], but its optimum level is still widely debated [5].

The prone position (PP) may also be part of the ventilatory strategy. This method was proposed more than 30 years ago, initially in pathophysiological studies [6,7]. Recently, Sud *et al*. [8] suggested, on the basis of pooled data from randomized, controlled trials, that PP may improve survival in the subgroup of patients with the most severe ARDS, that is, those with a ratio of partial pressure of arterial O_2 _to fraction of inspired O_2 _(PaO_2_/FiO_2_) < 100 mmHg. Many questions remain unresolved. In particular, response to PP is usually defined according to changes in PaO_2_, with responders being those in whom the PaO_2_/FiO_2 _ratio increases > 20 mmHg after one to six hours in the PP [9-11]. However, we have previously reported that PP allows recruitment of a slow compartment previously excluded from ventilation [12]. This was associated with a decrease in partial pressure of arterial CO_2 _(PaCO_2_), an indirect reflection of the reduction of the alveolar dead space (VD_alv_) [12]. Gattinoni *et al*. [10] also reported that the prognosis is improved in patients in whom PaCO_2 _declines after an initial PP session. Finally, VD_alv _appears to be an independent risk factor for mortality in patients with ARDS [13]. In a recent study, Siddiki *et al*. [14] proposed evaluating the physiological dead space fraction (VD_physiol_/V_T_) by using a rearranged alveolar gas equation for PaCO_2 _without any expired CO_2 _measurement.

In this context, we conducted a prospective physiological study to evaluate the impact of PP on ventilatory mechanics, gas exchange and VD_alv_. Our main objective was to validate our hypothesis that changes in PaCO_2 _and VD_alv _might be more relevant than changes in PaO_2 _in defining the respiratory response to PP. Our second objective was to validate the method of evaluation of the VD_physiol_/V_T _proposed by Siddiki *et al*. [14].

## Materials and methods

In our unit, patients with a PaO_2_/FiO_2 _ratio < 100 mmHg after 24 to 48 hours of mechanical ventilation are systematically turned to PP when hemodynamically stable [15]. Our study was approved by the Ethics Committee of the "Société de Réanimation de Langue Française" (SRLF-CE 07-213). After obtaining informed consent from the patients' relatives, 15 patients were included in the study between January 2008 and March 2010. Inclusion criteria were (1) the presence of ARDS according to the definition of the Acute Respiratory Distress Syndrome Network [3]; (2) persistence of severe hypoxemia after 48 hours of mechanical ventilation, defined as a PaO_2_/FiO_2 _ratio < 100 mmHg; and (3) hemodynamic stability, defined as systolic blood pressure > 90 mmHg with norepinephrine infusion at a rate < 0.5 μg/kg/minute. Patients with chronic obstructive pulmonary disease were excluded.

All patients were ventilated in volume-controlled mode (Servo-i; Maquet SA, Ardon, France), sedated and paralyzed by infusion of atracurium. The heat and moisture exchanger was routinely removed and replaced by a heated humidifier to reduce instrumental dead space as previously reported [16]. The ventilator settings included a "moderately restricted" V_T _of 6 to 8 mL/kg measured body weight, a respiratory rate allowing us to limit hypercapnia without generating intrinsic PEEP and an inspiration/expiration ratio of 1:2 with an end inspiratory pause of 0.5 seconds. Pplat was strictly limited < 30 cmH_2_O, and the PEEP selected was that which corrected the intrinsic PEEP, if any [17]. Ventilator settings were kept constant throughout the study. A recruitment maneuver was never used, and suction was not systematically performed. All patients were continuously monitored in terms of blood pressure with an arterial catheter, heart rate and O_2 _saturation by pulse oximetry.

The study was conducted during the first session of PP. Our sessions routinely last 15 to 18 hours per day. Blood gas analysis, Pplat, total PEEP, end-tidal CO_2 _(P_etCO2_) and mixed expired CO_2 _(P_ECO2_) were recorded with the patient in the supine position, just before turning the patient to the PP, and every 3 hours in the PP until 15 hours had elapsed. Expired CO_2 _was measured by a sensor positioned between the proximal end of the endotracheal tube and the Y piece of the ventilator circuit (COSMO; Novametrix, Wallingford, CT, USA). The ratio of VD/V_T _was calculated using the simplified Bohr equation [18] as follows: (1) VD_alv_/V_T _= 1 - P_etCO2_/PaCO_2 _and (2) VD_physiol_/V_T _= 1 - P_ECO2_/PaCO_2_.

The estimated VD_physiol_/V_T _ratio was calculated as 1 - [(0.86 × VCO_2est_)/(VE × PaCO_2_)], where VCO_2est _is the estimated CO_2 _production calculated using the Harris-Benedict equation [19] and VE is the expired minute ventilation.

Intrinsic PEEP was measured during a four-second end-expiratory occlusion period. Pplat was measured during a 0.5-second end-inspiratory pause. Respiratory system compliance (Crs) was calculated as Crs = V_T_/(Pplat - PEEP_total_). Responders to PP were defined in two different ways: (1) an increase in PaO_2_/FiO_2 _ratio > 20 mmHg after 15 hours of PP or (2) a decrease in PaCO_2 _> 2 mmHg after 15 hours of PP.

### Statistical analysis

Statistical analysis was performed using StatView 5 software (SAS Institute Inc., Cary, NC, USA). The continuous variables were expressed as medians (1st to 3rd interquartile range). Analysis of variance for repeated measurements was used for each parameter, and *P *< 0.05 was considered statistically significant. Measured VD_physiol_/V_T _and estimated VD_physiol_/V_T _were compared according to Bland-Altman analysis, together with the concordance correlation coefficient in 78 paired data. The same method was used to compare variations of measured and estimated VD_physiol_/V_T _every three hours while the patient was in PP.

## Results

Two patients were excluded from the study because of a history of severe chronic obstructive pulmonary disease, which left a study population of 13 patients. The patients' median age was 53 years (1st to 3rd interquartile range, 48 to 59 years), their median Simplified Acute Physiology Score II score was 62 (1st to 3rd interquartile range, 35 to 71) and their median Sequential Organ Failure Assessment score was 11 (1st to 3rd interquartile range, 8-13). All patients except one had ARDS of pulmonary origin. Eight patients had pneumonia, with six cases related to streptococcus pneumonia and two due to influenza (H1N1 virus). Two patients had aspiration, one had toxic shock syndrome and two had ARDS due to miscellaneous causes. No patient had abdominal hypertension or traumatic lung injury. Eleven patients required norepinephrine infusion. Respiratory parameters and blood gas analysis at the time of inclusion are reported in Table 1.

**Table 1:** Respiratory parameters and blood gas analysis at inclusion^a^

Parameters	Median	1st to 3rd interquartile range
LIS	3.25	3 to 3.25
Tidal volume, mL/kg IDB	6.2	5.6 to 8.3
RR, breaths/minute	22	18 to 26
PEEP, cmH_2_O	6	5 to 7
FiO_2_, %	90	90 to 100
Pplat, cmH_2_O	27	26 to 28
PaO_2_/FiO_2_, mmHg	70	51 to 77
PaCO_2_, mmHg	58	52 to 60
Crs, mL/cmH_2_O	16	13 to 30
VD_alv_/V_T_	0.42	0.35 to 0.47
VD_alv_, mL	159	95 to 236

^a^Crs: compliance of the respiratory system; IDB: ideal body weight; LIS: lung injury score [32]; PaCO_2_: partial pressure of arterial CO_2_; PaO_2_/FiO_2_: ratio of partial pressure of arterial O_2 _to fraction of inspired O_2_; PEEP: positive end-expiratory pressure; Pplat: plateau pressure; RR: respiratory rate; VD_alv_/V_T_: ratio of alveolar dead space to tidal volume.

A significant increase in PaO_2_/FiO_2 _ratio occurred after 15 hours of PP, from 70 mmHg (51 to 77) in the supine position to 99 mmHg in the prone (83 to 139) (*P *< 0.0001) (Table 2). A significant decrease in PaCO_2 _was also observed, from 58 mmHg (52 to 60) to 52 mmHg (47 to 56) (*P *= 0.04) (Table 2), with the lowest value occurring after nine hours of PP. As noted in Table 2, Pplat was significantly reduced (*P *= 0.0004) and Crs improved (from 16 mL/cmH_2_O (13 to 30) to 18 mL/cmH_2_O (15 to 30); *P *= 0.02). Finally, the VD_alv_/V_T _ratio was significantly reduced from 0.42 (0.35 to 0.47) to 0.40 (0.26 to 0.45), with the lowest value occurring after three hours in PP (hour 3) (0.31) (Table 2).

**Table 2:** Changes in respiratory mechanics, blood gas analysis and VD_alv _in PP

Parameters	Supine	PP H3	PP H6	PP H9	PP H12	PP H15	*P *value
PaO_2_/FiO_2_, mmHg	70 (51 to 77)	91 (81 to 103)	87 (73 to 139)	90 (81 to 111)	93 (83 to 137)	99 (83 to 139)	< 0.0001
PaCO_2_, mmHg	58 (52 to 60)	54 (51 to 58)	54 (45 to 59)	50 (47 to 59)	54 (47 to 56)	52 (47 to 56)	0.04
Pplat, cmH_2_O	27 (26 to 28)	25 (23 to 27)	25 (22 to 26)	25 (23 to 26)	25 (21 to 26)	25 (24 to 26)	0.0004
Crs, mL/cmH_2_O	16 (13 to 30)	18 (14 to 36)	17 (15 to 40)	18 (15 to 38)	19 (15 to 38)	18 (15 to 30)	0.02
VD_alv_/V_T_	0.42 (0.35 to 0.47)	0.31 (0.28 to 0.41)	0.35 (0.22 to 0.39)	0.35 (0.26 to 0.39)	0.39 (0.28 to 0.44)	0.40 (0.26 to 0.45)	0.007

^a^Crs: compliance of the respiratory system; PP: prone position, Pplat: plateau pressure, VD_alv_/V_T_: ratio of alveolar dead space to tidal volume. H3, H6, H9, H12 and H15: 3, 6, 9, 12 and 15 hours of PP, respectively. *P *value is between supine position and PP. Data are expressed as medians (1^st ^to 3^rd ^interquartile range).

**Figure 1:**
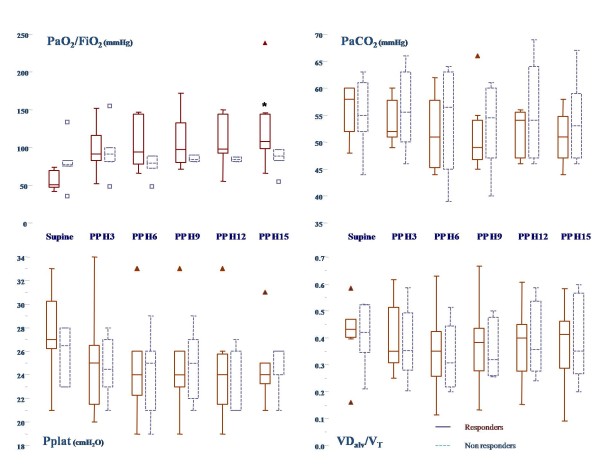
**Alterations during PP in PaO_2_/FiO_2_, PaCO_2_, plateau pressure (Pplat) and alveolar dead space (VD_alv_/V_T_) in responders (solid lines) and nonresponders (dotted lines) according to PaO_2_/FiO_2 _changes**. "PaO_2 _responders" were defined by an increase in PaO_2_/FiO_2 _> 20 mmHg after 15 hours of PP (PP H15). Shown are box and whisker plots. Median = horizontal line inside the box; upper and lower quartiles = whisker plot. Boxes and triangles represent values higher or lower than the upper or lower quartiles. *P < 0.05 for comparison of changes in responders versus nonresponders. PP: prone position.

**Figure 2:**
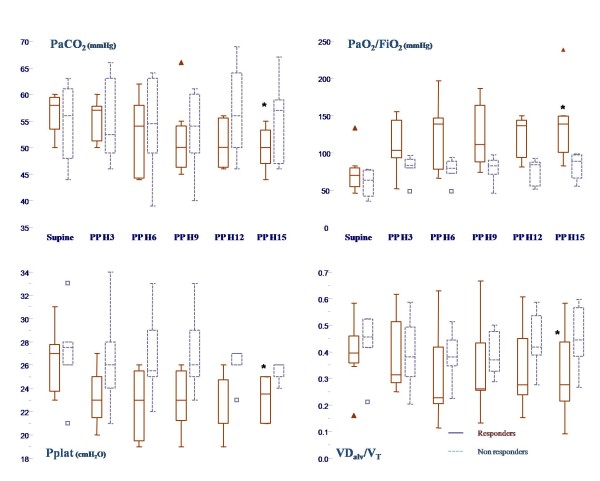
**Alterations during PP in PaO_2_/FiO_2_, PaCO_2_, plateau pressure (Pplat) and alveolar dead space (VD_alv_/V_T_) in responders (solid lines) and nonresponders (dotted lines) according to PaCO_2 _changes**. "PaCO_2 _responders" were defined by a decrease in PaCO_2 _> 2 mmHg after 15 hours of PP (PP H15). Shown are box and whisker plots. Median = horizontal line inside the box; upper and lower quartiles = whisker plot. Boxes and triangles represent values higher or lower than the upper or lower quartiles. *P < 0.05 for comparison of changes in responders versus nonresponders. PP: prone position.

**Figure 3:**
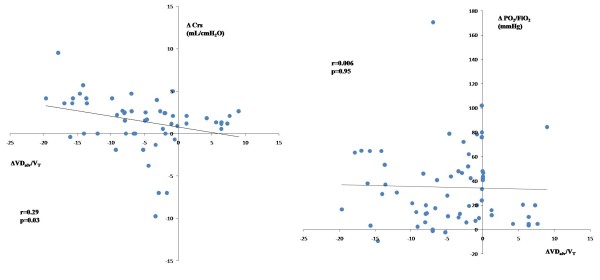
**Correlation between changes in alveolar dead space (ΔVD_alv_/V_T_) and changes in compliance of the respiratory system (ΔCrs, left) or in PaO_2_/FiO_2 _(ΔPaO_2_/FiO_2_, right) at each time of the study when compared with the supine position**.

Seven patients were classified as "PaO_2 _responders" and six were classified as "PaO_2 _nonresponders" according to PaO_2_/FiO_2 _ratio changes. No differences in VD_alv_/V_T _ratios or PaCO_2 _or Pplat alterations during PP were observed between groups (Table 3 and Figure 1), whereas Crs increased more in the responders (Table 3). Seven patients were also classified as "PaCO_2 _responders" and six as "PaCO_2 _nonresponders" according to the PaCO_2 _changes. However, when compared with the PaO_2_/FiO_2 _classification, four patients were classified differently. As shown in Table 4 and Figure 2, VD_alv_/V_T_, PaO_2_/FiO_2_, PaCO_2_, Pplat and Crs were significantly more altered in responders than in nonresponders. As shown in Figure 3, we found no correlation between changes in VD_alv_/V_T _and changes in PaO_2_/FiO_2 _(*P *= 0.95), whereas we found a negative correlation between changes in VD_alv_/V_T _and changes in Crs (*r *= 0.29, *P *= 0.03).

**Table 3:** Changes in respiratory mechanics, blood gas analysis and VD_alv _in PaO_2 _responders (*n *= 7) and PaO_2 _nonresponders (*n *= 6)^**a**^

		Supine		PP H3		PP H6		PP H9		PP H12		PP H15		
**Parameters**		**Median**	**1st to 3rd interquartile range**	**Median**	**1st to 3rd interquartile range**	**Median**	**1st to 3rd interquartile range**	**Median**	**1st to 3rd interquartile range**	**Median**	**1st to 3rd interquartile range**	**Median**	**1st to 3rd interquartile range**	***P *value**

PaO_2_/FiO_2_, mmHg	R	51	(48 to 69)	91	(86 to 112)	94	(83 to 142)	97	(86 to 126)	98	(93 to 142)	108	(99 to 142)	0.0003
	NR	77	(76 to 81)	91	(82 to 99)	79	(73 to 88)	84	(82 to 99)	84	(82 to 87)	89	(82 to 97)	
VD_alv_/V_T_	R	0.43	(0.41 to 0.47)	0.35	(0.31 to 0.46)	0.35	(0.29 to 0.41)	0.38	(0.23 to 0.42)	0.40	(0.31 to 0.40)	0.41	(0.32 to 0.45)	0.31
	NR	0.42	(0.36 to 0.50)	0.35	(0.28 to 0.47)	0.31	(0.22 to 0.43)	0.32	(0.27 to 0.44)	0.36	(0.28 to 0.51)	0.35	(0.27 to 0.53)	
PaCO_2_, mmHg	R	58	(54 to 60)	52	(51 to 58)	51	(47 to 57)	49	(48 to 53)	54	(48 to 55)	51	(47 to 55)	0.14
	NR	55	(52 to 60)	56	(51 to 62)	57	(48 to 62)	55	(48 to 60)	54	(48 to 63)	53	(48 to 58)	
Pplat, cmH_2_O	R	27	(27 to 30)	25	(22 to 26)	24	(23 to 26)	24	(23 to 26)	24	(22 to 26)	24	(24 to 25)	0.27
	NR	27	(24 to 28)	25	(24 to 28)	25	(22 to 26)	25	(23 to 27)	26	(22 to 26)	26	(25 to 26)	
Crs, mL/cmH_2_O	R	16	(13 to 28)	19	(16 to 37)	18	(16 to 38)	18	(16 to 35)	20	(17 to 35)	19	(17 to 33)	0.023
	NR	19	(14 to 31)	21	(14 to 33)	21	(14 to 36)	21	(14 to 34)	19	(15 to 34)	19	(15 to 34)	

^a^Crs: compliance of the respiratory system; NR: nonresponders; PP: prone position; Pplat: plateau pressure; R: responders; VD_alv_/V_T_: ratio of alveolar dead space to tidal volume. *P *values represent comparison of changes between responders and nonresponders. H3, H6, H9, H12 and H15: 3, 6, 9, 12 and 15 hours of PP, respectively. Responders are defined as patients whose PaO_2_/FiO_2 _increased > 20 mmHg after 15 hours of PP.

**Table 4:** Changes in respiratory mechanics, blood gas analysis and VD_alv _in PaCO_2 _responders (*n *= 7) and PaCO_2 _nonresponders (*n *= 6)^**a**^

		Supine		PP H3		PP H6		PP H9		PP H12		PP H15		
**Parameters**		**Median**	**1st to 3rd interquartile range**	**Median**	**1st to 3rd interquartile range**	**Median**	**1st to 3rd interquartile range**	**Median**	**1st to 3rd interquartile range**	**Median**	**1st to 3rd interquartile range**	**Median**	**1st to 3rd interquartile range**	***P *values**

PaCO_2_, mmHg)	R	58	(55 to 59)	57	(51 to 57)	54	(44 to 57)	50	(46 to 53)	50	(46 to 55)	50	(47 to 52)	0.005
	NR	56	(49 to 60)	52	(49 to 60)	54	(49 to 62)	54	(49 to 60)	56	(51 to 62)	57	(49 to 59)	
VD_alv_/V_T_	R	0.40	(0.37 to 0.45)	0.31	(0.29 to 0.46)	0.23	(0.31 to 0.40)	0.26	(0.26 to 0.42)	0.28	(0.24 to 0.44)	0.28	(0.23 to 0.43)	0.005
	NR	0.45	(0.42 to 0.51)	0.38	(0.32 to 0.47)	0.38	(0.35 to 0.43)	0.37	(0.33 to 0.45)	0.42	(0.39 to 0.51)	0.44	(0.39 to 0.54)	
PaO_2_/FiO_2_, mmHg	R	70	(59 to 78)	103	(96 to 136)	138	(83 to 146)	111	(91 to 156)	136	(95 to 142)	139	(103 to 148)	0.0001
	NR	63	(44 to 76)	83	(80 to 89)	79	(73 to 88)	83	(74 to 88)	84	(62 to 87)	89	(70 to 97)	
Pplat, cmH_2_O	R	27	(24 to 27)	23	(22 to 25)	23	(20 to 25)	23	(22 to 25)	21	(21 to 25)	23	(21 to 25)	0.002
	NR	28	(26 to 28)	26	(24 to 28)	26	(25 to 28)	26	(25 to 28)	26	(25 to 26)	26	(25 to 26)	
Crs, mL/cmH_2_O	R	28	(15 to 30)	30	(18 to 36)	34	(17 to 41)	32	(18 to 38)	32	(19 to 39)	31	(18 to 39)	0.002
	NR	15	(12 to 20)	15	(13 to 24)	15	(13 to 23)	15	(13 to 23)	15	(14 to 22)	15	(14 to 22)	

^a^Crs: compliance of the respiratory system; NR: nonresponders; PP: prone position; Pplat: plateau pressure; R: responders; VD_alv_/V_T_: ratio of alveolar dead space to tidal volume. *P *value represents comparison of changes between responders and nonresponders. H3, H6, H9, H12 and H15: 3, 6, 9, 12 and 15 hours of PP, respectively. Responders are defined as patients whose PaCO_2 _decreased > 2 mmHg after 15 hours of PP.

**Figure 4:**
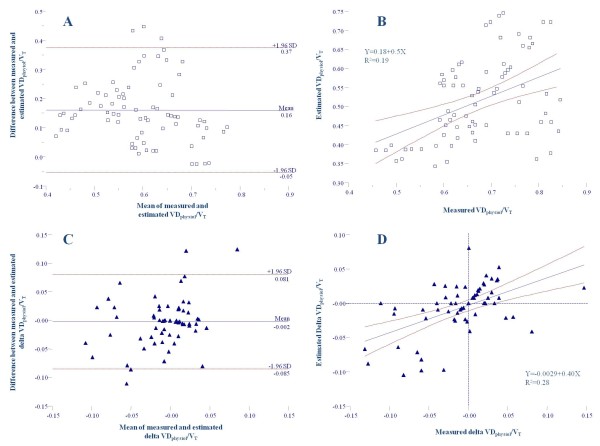
**Comparison between measured VD_physiol_/V_T _and estimated VD_physiol_/V_T _**[14]**using a Bland and Altman representation (left) and a linear correlation (right)**. **(A) **and **(B) **Comparison for each paired data set (*n *= 78) in the supine position and after 3, 6, 9, 12 and 15 hours in the prone position. **(C) **and **(D) **Comparison of changes in VD_physiol_/V_T _assessed according to the two methods between each time of measurement and the previous one. VD_physiol_/V_T_: ratio of physiological dead space to tidal volume.

As shown in Figure 4, estimated VD_physiol_/V_T _systematically underestimated measured VD_physiol_/V_T_, with a poor concordance correlation coefficient of 0.19 (95% confidence interval (95% CI) 0.091 to 0.28), a bias of 0.16 and an agreement between -0.05 and 0.37. Concerning changes in VD_physiol_/V_T _during PP, estimated VD_physiol_/V_T _had a concordance correlation coefficient of 0.51 (95% CI 0.32 to 0.66) (Figure 4).

## Discussion

One of the objectives of our study was to describe alterations in VD_alv _induced by PP. ARDS is characterized by a heterogeneous lung with the existence of a slow compartment [18,20], defined as areas available for, but partially or totally excluded from, ventilation due in part to a bronchiolar collapse [12,21]. In a previous study, we reported that PP may induce recruitment of this slow compartment, as suggested by its ability to counteract intrinsic PEEP and to decrease the expiratory time constant [12]. In the same study, we also reported that PP leads to a decrease in PaCO_2_, suggesting diminution of VD_alv _(alveolar dead space) [12]. Our present study demonstrates that PP may induce a decrease in VD_alv_. It occurred from the third hour and was maintained throughout the PP session. VD_alv _may be the consequence of nonperfused or poorly perfused lung areas in ventilated anterior areas, but also of a slow compartment partially excluded from ventilation. Our results suggest that PP induces functional lung recruitment, especially since decreases in VD_alv _related to PP were associated with a decrease in Pplat and strongly correlated with improvement in compliance. Interestingly, in a previous study of 16 ARDS patients, Pelosi *et al*. [22] did not find a decrease in VD_physiol _after 120 minutes in PP. One of the explanations for this discrepancy could be the different levels of PEEP in the two studies: 12.3 cmH_2_O in Pelosi *et al*.'s study and only 6 cmH_2_O in our study. However, Protti *et al*. [23], in a study of patients ventilated with a PEEP of 13 cmH_2_O, demonstrated a strong relation between lung recruitability and decreased PaCO_2 _related to PP. Pelosi *et al*. also did not report a decrease in Pplat in PP, as we found, but after returning patients to the supine position [22]. This could be explained by the fact that they used roll under the upper part of the chest wall, leading to a significant impairment in chest wall compliance [22], whereas we did not.

The most beneficial reported effect of PP is oxygenation improvement [24,25]. However, this better oxygenation can be due to (1) lung recruitment related to restoration of functional residual capacity [7] and improvement of the diaphragmatic movement in the posterior part [26-28] or (2) simply to an improvement in the ventilation/perfusion ratio due to a decreased hydrostatic gradient between the anterior and posterior parts of the lung [26,29]. Whereas the first mechanism is crucial, one can say that the second mechanism is less important. This is why the second objective of our study was to test whether the response to PP in terms of PaCO_2 _was physiologically more relevant than in terms of PaO_2_/FiO_2 _ratio. Gattinoni *et al*. [10] reported that an increase in PaO_2_/FiO_2 _ratio > 20 mmHg after six hours of PP is not predictive of the patient's prognosis, whereas a decline in PaCO_2 _≥1 mmHg is. In our present study, 7 of 13 patients were PaO_2 _responders (increased PaO_2_/FiO_2 _ratio > 20 mmHg after 15 hours of PP). However, changes in Pplat, PaCO_2 _and VD_alv _did not differ between PaO_2 _responders and PaO_2 _nonresponders. On the other hand, 7 of 13 patients were PaCO_2 _responders (decreased PaCO_2 _> 2 mmHg after 15 hours of PP). PaCO_2 _responders had a significant decrease in Pplat and VD_alv_, as well as a significant increase in oxygenation and compliance, compared with nonresponders. Our results are in accordance with a recent study of 32 ARDS patients [23], in which the investigators reported that PaCO_2 _variation induced by PP, and not PaO_2_/FiO_2 _variation, is associated with lung recruitability. Interestingly, in our study, changes in VD_alv _were not correlated with changes in oxygenation but were strongly correlated with changes in compliance of the respiratory system.

An unexpected result of our work concerns the change over time of respiratory mechanics, blood gas analysis and VD_alv_. For many years, our PP protocol has been to turn patients to PP for up to 15 to 18 hours per day for 3 days [15]. In the study by Mancebo *et al*. [30], which concluded that PP may reduce mortality in patients with severe ARDS, PP sessions lasted 20 hours/day. In a recent study, we demonstrated that PP sessions that lasted 18 hours/day were independently associated with survival [31]. In the present study, the maximum effect of PP for VD_alv_, PaCO_2 _and Pplat occurred six to nine hours after turning patients to PP. Later the effect seemed to be a decline. How this affects the effect of PP on patient prognosis remains to be elucidated.

The second objective of our study was to validate a recently proposed method to evaluate the VD_physiol_/V_T _ratio [14]. The method is based on CO_2 _production calculated from the Harris-Benedict equation [19] and on the expired minute ventilation. Siddiki *et al*. [14] reported that it was associated with mortality in acute lung injury patients in a dose-response manner and proposed its routine use to estimate VD_physiol_/V_T_. However, they did not report any comparison with measured VD_physiol_/V_T_. In the present study, we have demonstrated that this method significantly underestimates VD_physiol_/V_T_, rendering it not accurate enough to assess the degree of lung injury. Interestingly, changes in estimated VD_physiol_/V_T _during PP appeared better correlated with changes in measured VD_physiol_/V_T _and could be proposed in the future in this field. Siddiki *et al*. [14] proposed the method in the context of a much larger series than ours and in patients with less severe ARDS, rendering it difficult to draw any definitive conclusions.

Our work is limited by the small number of patients included. This is a consequence of our routine protocol, which strictly restricts PP to patients with the most severe ARDS, that is, those with a PaO_2_/FiO_2 _ratio < 100 mmHg after 48 hours of ventilation. This also explains why it is not possible to link our results to outcomes. However, despite this limitation, we consider our results relevant from a physiological point of view.

## Conclusions

In conclusion, our study demonstrates that PP induces a decrease in PaCO_2 _and VD_alv_. This is related to an improvement in respiratory mechanics, with a decrease in Pplat and an increase in compliance. Testing the response to PP appeared to be physiologically more relevant using PaCO_2 _changes than PaO_2_/FiO_2 _changes. How this may affect management at the bedside remains to be studied. Estimated VD_physiol_/V_T _ratios systematically underestimated measured VD_physiol_/V_T _ratios.

## Key messages

• PP induced a decrease in VD_alv_/V_T_, which was correlated with an improvement in respiratory mechanics.

• Defining the respiratory response to PP appeared more relevant when using PaCO_2 _changes rather than PaO_2_/FiO_2 _changes.

• Estimated VD_physiol_/V_T _using the Harris-Benedict equation systematically underestimated measured VD_physiol_/V_T_.

## Abbreviations

ARDS: acute respiratory distress syndrome; P_ECO2_: mixed expired PCO_2_; PEEP: positive end-expiratory pressure; P_etCO2_: end-tidal PCO_2_; PP: prone position; Pplat: plateau pressure; VD_alv_: alveolar dead space; VD_physiol_: physiological dead space.

## Competing interests

The authors declare that they have no competing interests, except that of receiving funds from Maquet SA (Ardon, France) to support the cost of publication.

## Authors' contributions

CC contributed to the acquisition of data, performed the data analysis, participated in the design of the study and the interpretation of the data, and wrote the manuscript. XR contributed to the acquisition of data, performed the data analysis and participated in the design of the study and the interpretation of the data. KB, SC, VC and BP contributed to the acquisition of data. AVB performed the data analysis, participated in the design of the study and the interpretation of the data, and wrote the manuscript. FJ participated in the design of the study and the interpretation of the data. All authors read and approved the final manuscript.
